# Improved deconvolution of natural products’ protein targets using diagnostic ions from chemical proteomics linkers

**DOI:** 10.3762/bjoc.20.199

**Published:** 2024-09-12

**Authors:** Andreas Wiest, Pavel Kielkowski

**Affiliations:** 1 LMU Munich, Department of Chemistry, Butenandtstr. 5-13, 81377 Munich, Germany

**Keywords:** chemical proteomics, diagnostic ions, mass spectrometry, target identification

## Abstract

Identification of interactions between proteins and natural products or similar active small molecules is crucial for understanding of their mechanism of action on a molecular level. To search elusive, often labile, and low-abundant conjugates between proteins and active compounds, chemical proteomics introduces a feasible strategy that allows to enrich and detect these conjugates. Recent advances in mass spectrometry techniques and search algorithms provide unprecedented depth of proteome coverage and the possibility to detect desired modified peptides with high sensitivity. The chemical ‘linker’ connecting an active compound–protein conjugate with a detection tag is the critical component of all chemical proteomic workflows. In this review, we discuss the properties and applications of different chemical proteomics linkers with special focus on their fragmentation releasing diagnostic ions and how these may improve the confidence in identified active compound–peptide conjugates. The application of advanced search options improves the identification rates and may help to identify otherwise difficult to find interactions between active compounds and proteins, which may result from unperturbed conditions, and thus are of high physiological relevance.

## Introduction

Natural products (NPs) have been pivotal for the development of modern medicine accompanying humans from the prehistorical age [1]. In the last century, NPs became the main source of the active scaffolds for the pharmaceutical industry with focus on principle of one compound, one target, and one disease [2–3]. In parallel, mass spectrometry (MS) has been crucial in many areas centered around the characterization of NPs [4]. First to annotate their often complex structures using diverse fragmentation techniques [4]. Nowadays, MS is applied for the identification of NPs’ cellular protein targets via MS-based chemical proteomics [5–6]. In proteomics and its sub-field of chemical proteomics, there are two major areas of development, mass spectrometry instrumentation and bioinformatics. The MS instruments acquire the mass spectra with significantly increased throughput and sensitivity. Acquisition techniques benefit from possible multistage ion separation and isolation, and diverse fragmentation techniques. All of that is nowadays combined in one instrument to provide greater flexibility of the spectra acquisition setup [7–16]. Given the sheer amount of data acquired by LC–MS/MS runs and the high density of information content in the MS spectra, another area that experienced progressive development includes search algorithms, which aim to maximize the information obtained from the acquired mass spectra and hence to improve rate and confidence of hit identifications [17–22].

**Figure 1:**
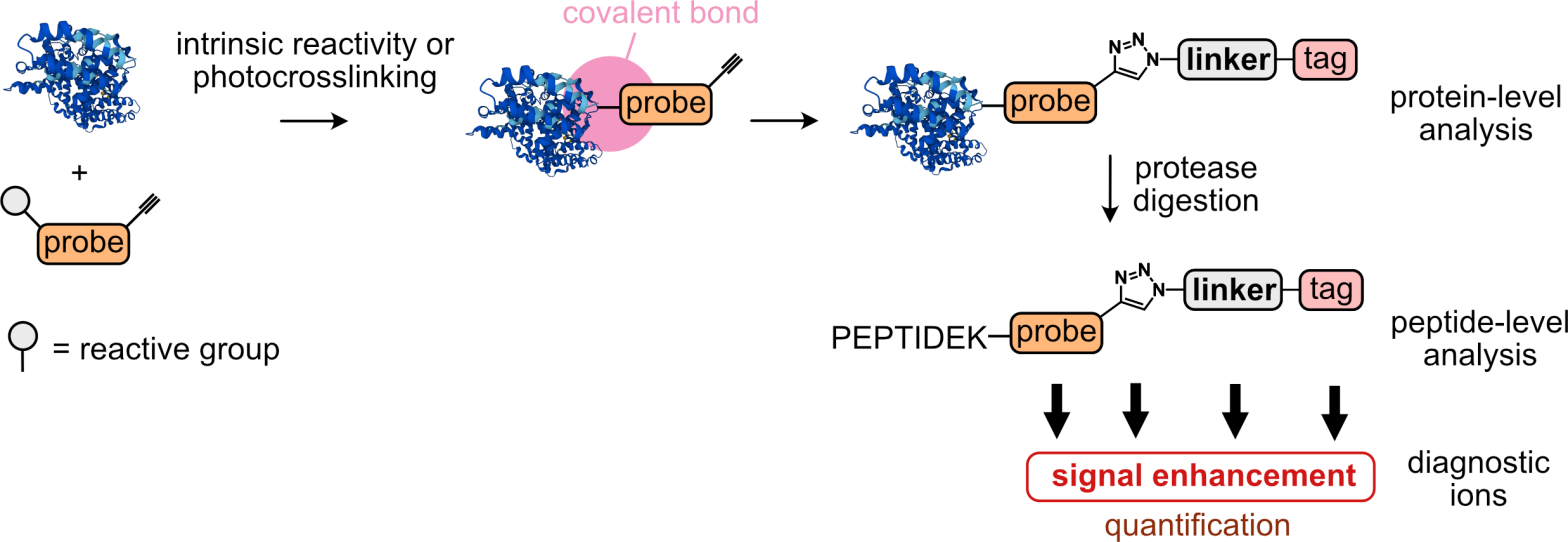
Overall chemical proteomics strategy to identify protein targets of natural products (NPs) and similar active small compounds. The example protein (blue) is an AlphaFold v2.0-generated prediction of bovine serum albumin (BSA) [23–24].

**Figure 2:**
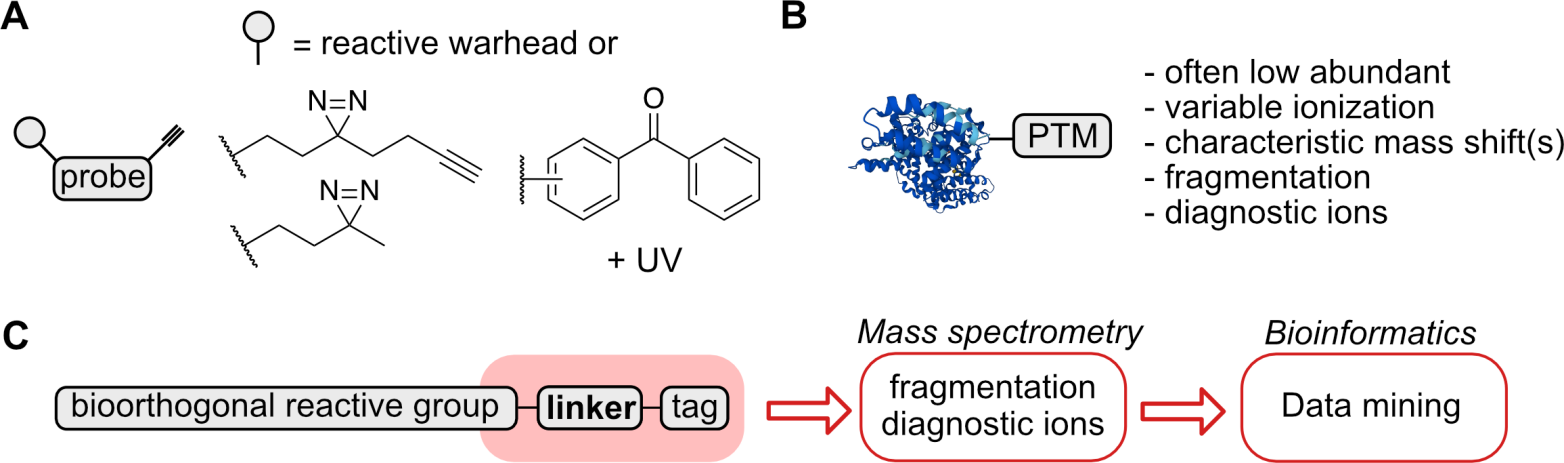
A) Design of mostly used photo-crosslinking groups. B) Mass spectrometry properties of proteins PTMs, which relate to probe–protein conjugates. C) General concept utilizing linkers to improve searches for protein–probe interactions.

All parts of the chemical proteomics experimental procedure, the sample preparation together with MS measurement and evaluation can be performed in a streamlined or automated way using sophisticated pipelines [13,17,21,25–27]. These are highly optimized to provide the desired protein coverage and robustness. Still, the critical part of the process starts with the careful design of a NP analogue and selection of the proper biological system, which may include animal models, 3D tissue-organoids, or 2D cell culture. Depending on the project aim, which typically might be to obtain the pathologically relevant protein targets or off-targets, this may further lead to optimization of the NP scaffold to improve the desired activity. In this review, we focus on the most frequently used chemical proteomic strategies that necessitate a covalent bond between a target/off-target protein and a tested NP or a similar active small compound (Figure 1). The covalent bond serving this purpose can be formed in two distinct ways: either the NP or small compound already contains a reactive, often electrophilic group directly or the reactive group, for example a photo-crosslinker, is attached synthetically to the basic scaffold (Figure 2A) [28–32]. However, this requires a structure–activity relationship (SAR) screening to ensure that the selected NP retains biological activity after modification. The term probe is further used to unite such NPs and active compounds. Furthermore, from this perspective, the formed probe–protein covalent conjugate shares many features with naturally occurring protein post-translational modifications (PTMs) offering productive exchange of the techniques and experimental setups used in both fields (Figure 2B) [33–34]. The second prerequisite required for a probe used in a chemical proteomic study is an embedded bioorthogonal handle, for example a terminal alkyne or azide, which is able to react chemoselectively with a tag facilitating unambiguous identification by a selected analytical technique, for example LC–MS/MS (Figure 1) [35–37]. A covalent bond between a probe and a protein is needed because of denaturing conditions used during the analysis. In contrast, relying on a noncovalent interaction would result in loss of the probe–protein interaction. The bioorthogonal handle is therefore essential for an enrichment or distinction of the probe–protein complex from the background. There are two levels on which the probe conjugates can be identified: either on the protein level as intact probe–protein conjugates or on the peptide level after protease digestion as a probe–peptide conjugate (Figure 1). Although the increased speed of acquisition of mass spectra leads to ever better coverage of proteomes submitted for analysis, the throughput has not yet reached the point in which all peptides and their modified forms can be identified and characterized in a single experiment. Therefore, an enrichment of the probe-modified peptides or enhancement of the signal-to-noise ratio is necessary for successful analysis (Figure 1). The future challenge to complete the chemical proteomic analysis will include the absolute quantification of those identified NP–peptide conjugates to estimate protein occupancy (Figure 1). Although mass spectrometry-based chemical proteomics represents a robust method for the identification of NP protein targets, in some cases, the synthetic effort associated with the above-described SAR study to find a functional NP probe might outweigh the advantages of this approach. Considering a complementary strategy that does not necessitate for NP modification might be desirable. This may include hydrogen–deuterium exchange mass spectrometry (HDX-MS), limited proteolysis-coupled mass spectrometry (LiP–MS), thermal proteome profiling (TPP), cellular thermal shift assay (CETSA), affinity selection-mass spectrometry (AS-MS), co-fractionation mass spectrometry (CF-MS), or mass spectrometry integrated with equilibrium dialysis for the discovery of allostery systematically (MIDAS) [38–44]. The chemical proteomics approach and related activity-based protein profiling has been reviewed comprehensively in several reviews [45–48]. Applications of cleavable linkers, which might be considered as pioneers of MS fragmentation-based platforms to search peptide-linker remainders, were reviewed thoroughly as well [49–51]. Recent advances in mass spectrometers technology and analysis pipelines enable to obtain more information from already established workflows, because they are becoming practically feasible only now. These will be discussed further in this review. We primarily focus on the linkers utilized for enrichment and identification of the probe–protein conjugates resembling probe–protein interaction in native conditions in living cells. Taken together, the linkers used in chemical proteomics workflows are applied broadly in many research areas including screening of fragment libraries, covalent modifiers, protein PTMs, protein–protein interactions, protein–nucleic acid interactions, and indeed NP–protein interactions, but they share many common features, which are of interest of this review (Figure 2C) [5,52]. These chemical linkers bridge an imaginative gap between the crude, highly complex, and structurally diverse cell or tissue lysates containing unknown probe–protein conjugate(s) and the desired analytical output with the information about the protein and conjugate identity. Thus, the linkers have not only the function to simply connect the probe scaffold with an affinity or reporter tag, but they carry previously underappreciated features enhancing the capabilities of nowadays mass spectrometric hardware and bioinformatic software tools.

## Review

### Principles of chemical proteomics workflows

**Figure 3:**
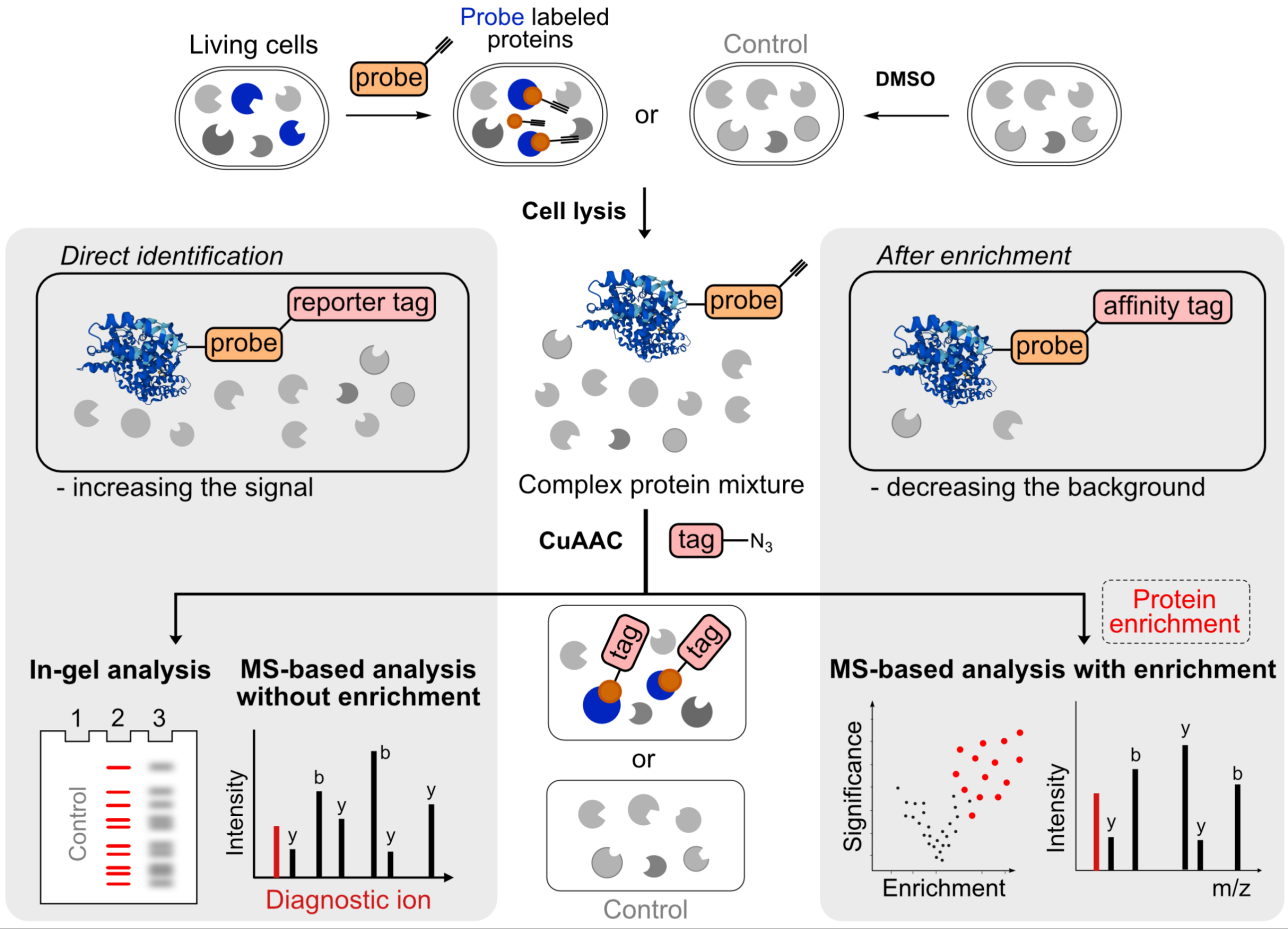
Direct and indirect approach to identify protein targets and representative chemical proteomics workflow visualizing the main steps; cells incubation with the probe, bioorthogonal reaction between probe–protein conjugates and a tag followed by analysis of those labeled proteins.

**Figure 4:**
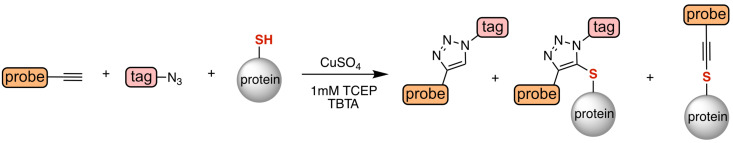
Products of the CuAAC side reactions.

The linker connects the affinity or reporter tag to the probe–protein conjugate to offer several principally different analytical pathways (Figure 3) [53–54]. Although chemical proteomics protocols may have many steps and operations in common, there is a great variability in how these individual steps are arranged into a workflow (Figure 3). The important aspect of any chemical proteomics study is the efficient coupling between the probe–protein conjugate and the selected linker bearing an affinity or reporter tag. To carry out this bioorthogonal reaction well-known chemistries were developed including traceless Staudinger ligation, Cu-catalyzed azide–alkyne cycloaddition (CuAAC), strain-promoted azide–alkyne cycloaddition (SPAAC), inverse electron-demand Diels–Alder reaction (IEDDA), and recently, azomethine imine (AMIs)–isonitrile ligation [37,55–60]. The kinetics, chemoselectivity, stability, and steric demand of the bioorthogonal tag attached on the probe are decisive factors during the selection procedure [61–62]. The most commonly used strategy is CuAAC due to its rapid reaction kinetics, robustness, and relatively small steric hindrance of the terminal alkyne, which is usually attached to the probe core scaffold to form an alkyne probe [5,63]. Once the covalent bond between the probe and protein is formed, the cells are lysed, and the probe–protein conjugates are reacted via CuAAC with an azide tag. While the CuAAC has been employed in various studies and has had large impact on many biological discoveries, an unspecific reactivity was often reported [54,64]. Recently, we described this background-forming reaction, which is based on an interrupted CuAAC mechanism [65]. The thiotriazole product of this reaction, which is indistinguishable in the protein-level downstream analysis, is formed by coupling between protein free thiol groups and the triazole–copper adduct (Figure 4). However, its formation can be avoided by eliminating the free alkyne probe from the CuAAC reaction mixture or by increasing the concentration of the reducing agent such as tris(2-carboxyethyl)phosphine (TCEP). The reversed chemical proteomics approach in which the azide probe and alkyne tag are used suffers from similar unspecific reactivity of the terminal alkyne with free thiols, including the protein cysteines reacting to thioalkyne conjugates [66–67]. This is caused by the relatively high concentration of the alkyne tag which is used to accelerate the reaction.

**Figure 5:**
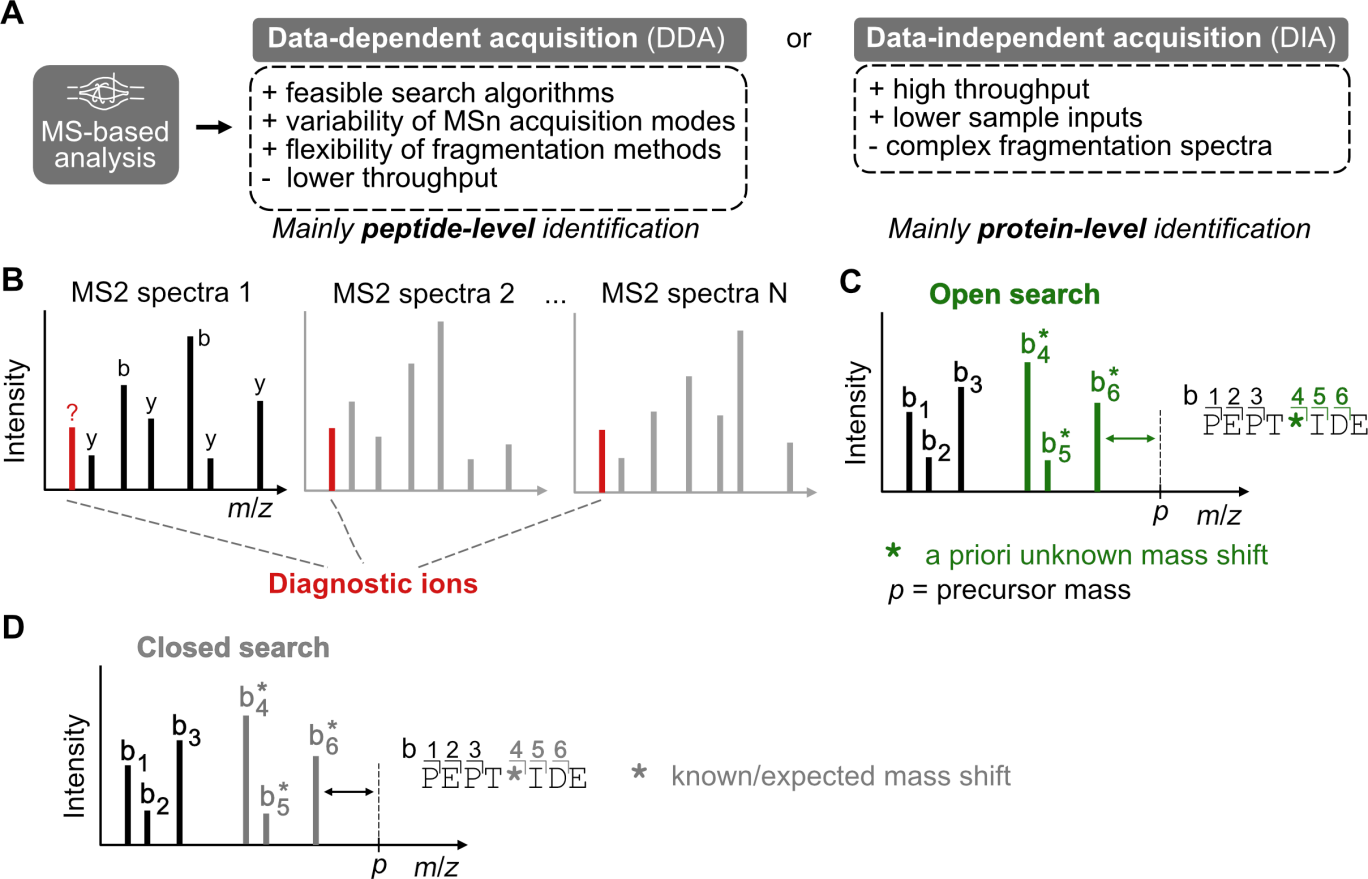
Search possibilities on peptide-level characterization. A) Comparison of DDA and DIA techniques. B) Diagnostic ions mining. C) Open search. D) Closed search.

The chemical proteomics workflows can be differentiated based on their read out, which can be done on the protein or peptide level (Figure 1). The protein-level identification leads to determination of protein(s) interacting with the probe. This might be the more straightforward approach as it requires smaller amounts of protein to begin with and typically the protein identification relies on standard and well-established peptide fragmentation used in the standard MS-based proteomics. Furthermore, the advantage of significantly improved data completeness using the data-independent acquisition (DIA), which in comparison to the previously applied data-dependent acquisition (DDA), achieves a higher number of protein identifications in relatively shorter time (Figure 5A) [68–69]. The mass spectra can be searched by standard proteomics search engines incorporated into freely available tools such as FragPipe platform, MaxQuant, and DIA-NN [17–18,20,70]. However, the probe-modified peptide and amino acid remain unknown. In contrast, the peptide-level identification is focused on the characterization of the actual probe-modified peptides. It might be more challenging as a larger protein amount to start with is necessary and the fragments of the probe-modified peptides may not be trivial to elucidate. The site identification is then reconstructed based on multiple rounds of searches utilizing different setups which may include labile searches with diagnostic ion mining, open and closed searches (Figure 5B–D) [19,71–74]. Exemplarily, the FragPipe workflows describing how to set up labile, open and closed searches and how we recalculated the published MS data for different linkers in this review can be downloaded from the publicly available database (PRIDE data set identifier PXD043402) [65]. Furthermore, the data processing can be adjusted and paralleled accordingly using orthogonal fragmentation techniques [75–76]. Because of the often low number of identified peptides bearing a desired modification, it is difficult to control the false-discovery rate (FDR) of the hits, and thus, the introduction of proper negative controls is of the upmost importance.

Thus far, the linkers were proposed and designed to mainly facilitate the enrichment of the probe–protein or probe–peptide conjugates and their selective release after enrichment. In this review we would like to highlight the fragmentation properties of the different linkers and resulting ions as the valuable source of information steering the site identifications and increasing the confidence in those identifications in MS-based chemical proteomics.

### In-gel chemical proteomics

**Figure 6:**
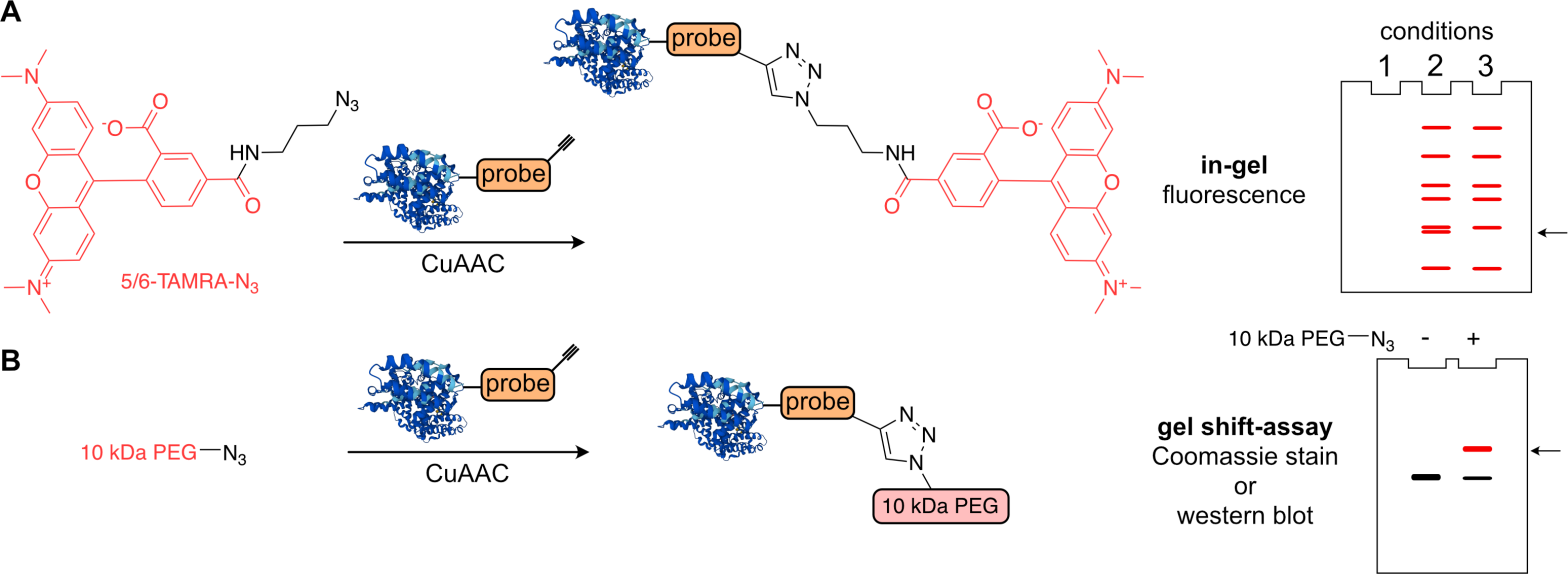
In-gel analysis using a tag with fluorophore (A) or via shift-assay (B).

The detection of the probe–protein conjugate(s) is based on the enhanced signal stemming from the probe–protein conjugate above the background from total protein present in the lysate. The detection can be done in several different ways. To obtain an overview of how many proteins are labeled in total and what is the intensity of the labeling, the probe–protein–tag conjugates are separated on sodium dodecyl sulfate polyacrylamide gel (SDS-PAGE) and visualized by in-gel fluorescence scanning (Figure 6A). In this approach the tag is a suitable fluorophore. Previously, during an introduction period of a chemical proteomics workflow of activity-based protein profiling (ABPP), the resulting fluorescent bands might be simply cut out from the gel and subjected to MS analysis to identify the labeled proteins [77–78]. The in-gel analysis is often used for target validation with corresponding protein mutants or knockout cell lines, with loss of the identified probe–protein interaction leading to disappearance of the fluorescence band from the SDS-PAGE. This approach also accounts for the protein-level identification; the exact modification site remains unknown. Here we show an example of the most used and commercially available rhodamine-derived dye (TAMRA-azide, Figure 6A). The bioorthogonal reaction between probe-labeled proteins and the TAMRA-azide yields a mixture of labeled and unlabeled proteins. The proteins are then separated on SDS-PAGE by their molecular weight and fluorescence of the labeled proteins is recorded (Figure 6A). Due to the high sensitivity of fluorescence read-out, this allows to identify protein labeling occurring already at sub-stoichiometric ratios. However, by in-gel analysis, it is not possible to quantify the extent of labeled proteins. To ensure the fidelity of the bioorthogonal reaction a control experiment in which the probe treatment is replaced by a plain solvent used to prepare the stock solution of the probe is necessary to identify any unspecific bands stemming from side reactivity of the azide–protein conjugate. In case a photo-crosslinker is used, another control experiment needs to be included, in which the cells/lysate are treated with the probe but not UV-irradiated. The non-covalent probe–protein interactions are not detected because of the denaturing conditions of the SDS-PAGE. Alternatively, the fidelity of the probe labeling can be tested by a competition experiment, in which the parent compound lacking a bioorthogonal tag is used at increasing concentration to outcompete the probe–protein interaction leading to disappearance of the fluorescent bands.

A gel shift assay is a complementary strategy to validate the labeling of a specific protein (Figure 6B) [65,79]. The assay is based on an increased retardation of the probe-labeled protein on SDS-PAGE due to the addition of a large tag, which significantly changes the molecular weight of the given protein. The shift on the gel can be then visualized by protein-specific antibody or coomassie staining. The linkers applied to perform the gel shift assay contain a linear PEG polymer with an approximate size ranging from one to dozens of kilodaltons. The main advantage of the gel shift assay is to obtain quantitative information of the protein’s probe modification extent, considering high efficiency of the click reaction. Given the bulkiness of the PEG–azide linker, the bioorthogonal reaction needs some optimization, including increased equivalents of the linker, elevated temperature, and/or longer reaction time. The limiting factor of this approach might be the size of the target proteins, because for larger proteins it might be more challenging to obtain the desired significant difference between mobility of probe-modified and unmodified protein.

### MS-based chemical proteomics without enrichment

**Figure 7:**
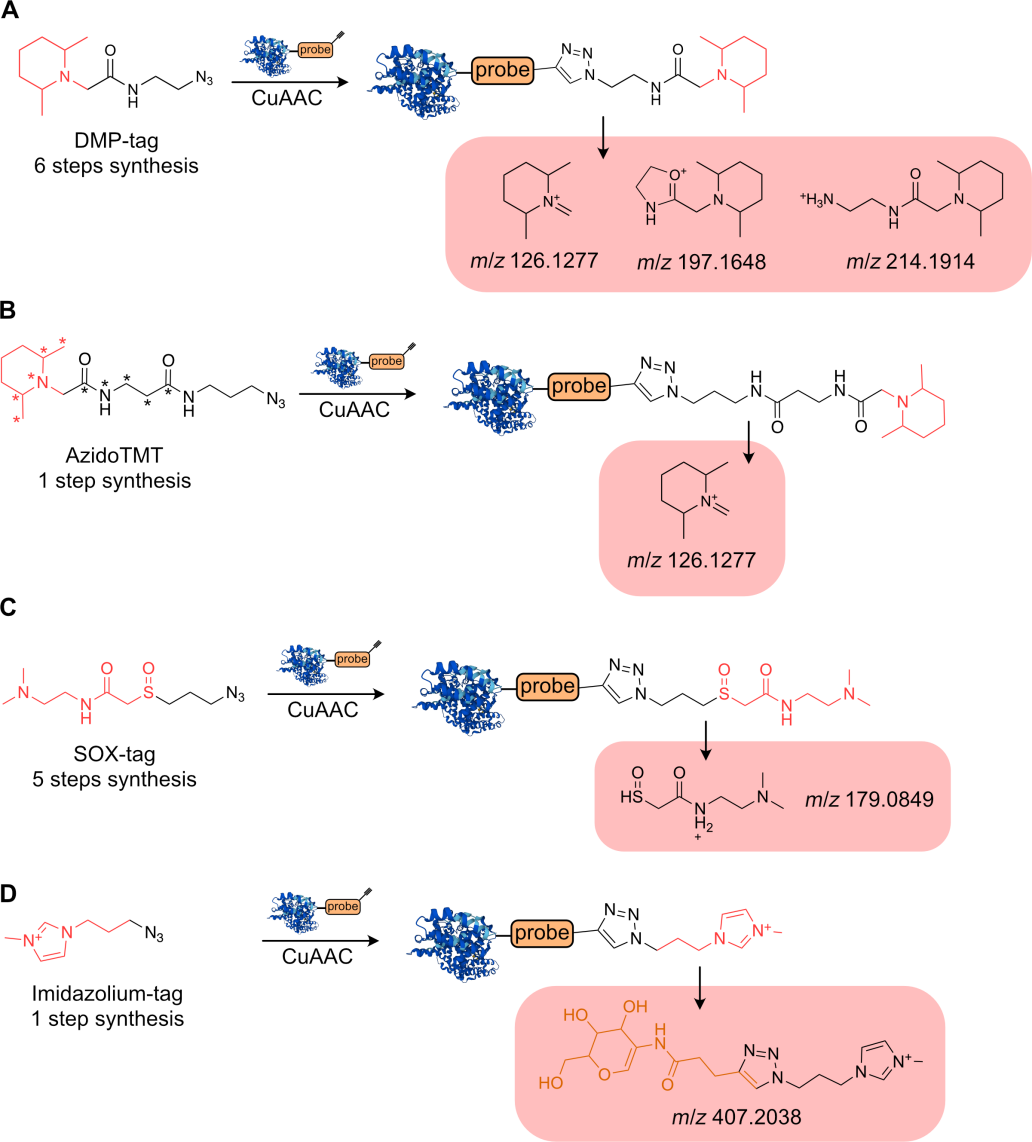
Reporter linkers. A) DMP-tag. B) AzidoTMT tag. C) SOX-tag. D) Imidazolium tag. *A star indicates the possible introduction of stable isotopes for MS quantification.

The aim of this approach is to isolate and characterize the probe–peptide conjugate and hence to provide direct evidence of the probe–protein interaction [80]. In principle, the direct characterization of the probe–peptide conjugate can be attempted, in which the coupling of the probe–peptide conjugate with a suitable reporter tag may help to improve the detectability in an excess of probe-unmodified peptides [65,81–82]. The speed of acquisition of mass spectra of the current LC–MS/MS methods does not ultimately cover all unmodified and modified peptides resulting from the proteolytic digest of a whole proteome. In other words, the complexity of the whole proteome digest is beyond the coverage and sensitivity of today’s mass spectrometers. On the other hand, the enrichment of the probe–peptide conjugates necessitates larger amounts of starting protein and application of often time-demanding and tedious workflows. Thus, the chemical tools increasing the signal intensity of the modified peptide species over the majority of background signals are of utmost interest and may bridge the gap between an ultimate characterization of complete proteome and enrichment-based methods. There are several chemical proteomics linkers reported that improve ionization of the probe–peptide conjugates or release a reporter ion during fragmentation or both (Figure 7). The reporter ion is important as it may serve two purposes. First, it facilitates the better identification of the modified peptides by search algorithms and second, if labeled with stable isotopes, it allows for quantification of the modified probe–peptide conjugates. Inspired by tandem mass spectrometry tags (TMT-tags) Makarov et al. have developed a ‘clickable’ 2,6-dimethylpiperidine analogue, the DMP-tag, which in contrast to standardly used isotopic tags bearing the *N*-hydroxysuccinimide ester (NHS-ester) reacting with all primary amines of lysine side-chains, allows to selectively label the probe–protein conjugates (Figure 7A) [81,83]. The DMP-tag can be prepared in a six-steps synthesis. The higher-energy collisional dissociation (HCD) releases the characteristic reporter ion at *m*/*z* 126.1277. In case the electron-transfer dissociation (ETD) is used the DMP-tag yields the reporter ion at *m*/*z* 114.1275. Of note, although ETD is more selective towards fragmentation of the peptide backbone leaving the side-chain modifications largely intact, the generation of the fluoranthene anion used for fragmentation slows down the fragmentation process and hence decreases the total amount of fragmented peptides and throughput of the LC–MS/MS measurement. To balance the throughput and fragmentation performance, the combination of HCD and ETD can be used in a single MS measurement. In such methods ETD is only triggered by probe-specific diagnostic ions released by the faster HCD fragmentation with the aim to fragment the same parent ion again with ETD to obtain a better fragmentation spectrum [84–85]. Similar to the DMP-tag, the AzidoTMT introduced by Ma et al. contains an azide to selectively label probe–peptide conjugates, but the synthesis takes advantage of the commercially available isotopically labeled 11plex-TMT (Figure 7B) [82]. The 11plexTMT reagent is reacted in one step with 3-azido-1-propanamine to give the desired AzidoTMT linker. Furthermore, Makarov et al. developed a linker containing a sulfoxide (SOX) group, which is well-known for specific fragmentation releasing the reporter ion at *m*/*z* 179.0849 (Figure 7C) [81,86–87]. The preparation of the tag is based on a five-step synthesis. However, in comparison to the DMP-tag or AzidoTMT the release of the reporter ion from the SOX-tag has somewhat lower intensity.

Calle et al. designed, synthesized, and validated a series of ‘clickable’ linkers for characterization of protein *O*-glycosylation containing positively charged pyridinium or imidazolium residues (Figure 7D) [88]. The presence of a stable positively charged moiety in the linker has, in case of imidazolium, increased by 70–90% the relative abundance of modified peptides. In combination with release of the glycan-related oxonium ions during fragmentation, such approach may improve the coverage of modified peptides. Taken together, the ever faster and more sensitive MS instruments in recent years drastically improved the proteome coverage of the LC–MS/MS methods to provide great insight into proteomes and enabled direct characterization of abundant modified proteins. The linkers releasing the reporter ion may serve as an interesting tool during transition from enrichment-based methods to direct ‘linker-free’ identification MS methods of desired probe–peptide conjugates.

### Enrichment-based chemical proteomics

**Figure 8:**
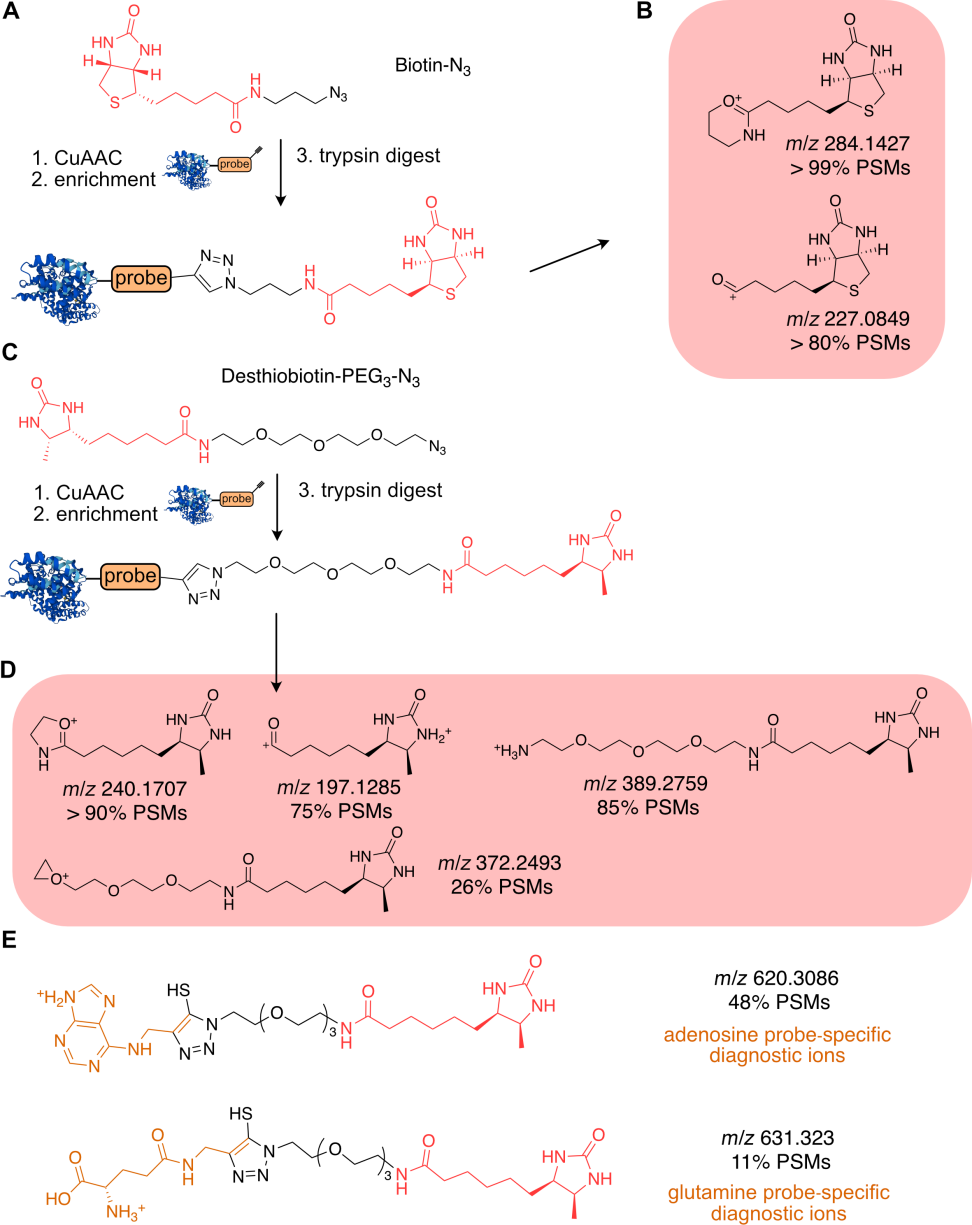
Biotin and desthiobition-based sample linkers and their associated diagnostic peaks. A) Structure of the biotin–azide linker and the CuAAC product. B) Diagnostic peaks derived from the biotin–azide linker. C) Structure of the desthiobiotin–PEG–azide linker and the corresponding CuAAC product. D) Diagnostic peaks resulting from fragmentation of the desthiobiotin–PEG–azide linker. E) Probe-specific diagnostic peaks after fragmentation of probe–peptide conjugates enriched using the desthiobiotin–PEG–azide linker.

The pursuit to decrease the complexity of proteomic samples led to development of numerous enrichment strategies using a variety of linkers as outlined below. The enrichment of the probe–protein or probe–peptide conjugates reduces the number of total peptides to be resolved by LC–MS/MS. The most common strategy to identify proteins interacting with a probe either directly in living cells or in cell lysates utilizes a linker with biotin (Figure 8A). This approach led for example to the identification of protein targets of the synthetic pyrethrin analogues cerulenin, hypothemicin, fimbrolide, and eupalmerin acetate [89–92]. The biotin forms a tight non-covalent complex with a glycoprotein called avidin or its non-glycosylated form streptavidin, from which the latter shows less of an unspecific protein–protein interaction due to missing glycan groups [93]. Streptavidin-coated paramagnetic agarose or acrylamide beads are used for pull-down of the probe-modified proteins [94]. The biotin-containing linker is introduced into the probe–protein conjugate via a selective bioorthogonal reaction. The main advantage of the biotin-based linkers is the possibility to apply stringent wash steps, due to the almost irreversible non-covalent complex formation between biotin and streptavidin (*K*_d_ ≈ 1 × 10^−14^ M) in aqueous solution. Another advantage is that after the enrichment and proteolytic digestion of each of the enriched proteins likely releases several suitable peptides for LC–MS/MS analysis resulting in a reliable identification of protein targets and relative quantification [95–97]. On the protein level, the biotin–azide linker was used to determine many probe–protein targets [92,98–100]. However, the modified probe–peptide conjugates linked with biotin mainly remain in complex with the streptavidin beads, which restricts the site identification [101]. The difference in protein intensities from the probe and control experiments can be visualized using a volcano plot or a waterfall plot and is also referred as protein-level experiment. Next, the elution of a desired biotin–probe–peptide conjugate can be carried out by high percentage of organic solvent, for example 80% acetonitrile. Yan et al. showed that such biotin–probe–peptides afforded characteristic fragmentation of the biotin linker, thus improving the identification of probe–peptide conjugates in cysteine-profiling experiments [102]. The biotin linker yields after HCD fragmentation a set of diagnostic ions including the prominent oxonium-biotin and dehydrobiotin ions (Figure 8B). Importantly, the oxonium-biotin fragment was found in more than 99% of the assigned spectra, so-called peptide spectrum matches (PSMs). The second most frequent diagnostic ion was the dehydrobiotin ion with occurrence in >80% of the spectra. The detailed list of all identified biotin and cysteine reactive probe related ions can be found in reference [102]. A plethora of biotin–azide linkers is commercially available, with the main difference lying in the length or presence of the PEG moiety, which has likely only minimal influence on the diagnostic peaks’ generation. Taken together, consideration of the oxonium-biotin and dehydrobiotin diagnostic ions increases the confidence in the identified probe–peptide conjugates.

Next, selective elution of the probe–peptide conjugates may be carried out using desthiobiotin (DTB) as affinity tag instead of biotin [103–104]. The DTB-tag allows to elute probe–peptide–DTB conjugates from streptavidin beads using a mixture of water, organic solvent – typically acetonitrile (ACN) – and an acid such as formic acid. The desthiobiotin–azide (DTB–azide) linker still possesses strong binding to streptavidin (*K*_d_ ≈ 1 × 10^−11^ M), but it can be released by mild washing conditions with organic solvent and low pH (Figure 8C) [105–106]. Similarly, desthiobiotin releases upon HCD fragmentation a set of characteristic diagnostic ions including oxonium-desthiobiotin, dehydrodesthiobiotin, and several other ions containing the PEG moiety (Figure 8D). The occurrence of these diagnostic ions ranges from >90% for oxonium-desthiobiotin and dehydrobiotin to ≈60% for the other presented diagnostic ions. During the studies of CuAAC side reactivity Wiest et al. proceeded with detailed characterization of desthiobition-linker-derived diagnostic ions and probe-specific fragments [65]. The latter are relevant for the discovery of probe–peptide conjugates as the fragments contain both desthiobiotin and probe-derived moieties (Figure 8E) [65]. In the study several small molecule probes were used including propargylamine, a glutamine derivative, *N*^6^-propargyladenosine and a tyrosine derivative to document the thiotriazole formation during CuAAC. The two selected examples of adenosine and glutamine-derived diagnostic ions are shown in Figure 8E. Since, in this case, the cysteine thiols were derivatized and the cysteine C–S bond was previously described to be susceptible to fragmentation, the fragments containing the thiol provide the information on the structure of the modified amino acid side chain [107–108]. Although the study was focused on the characterization of the side-products of CuAAC, it shares multiple features applicable for determination of NP–peptide conjugates in complex proteomic samples.

**Figure 9:**
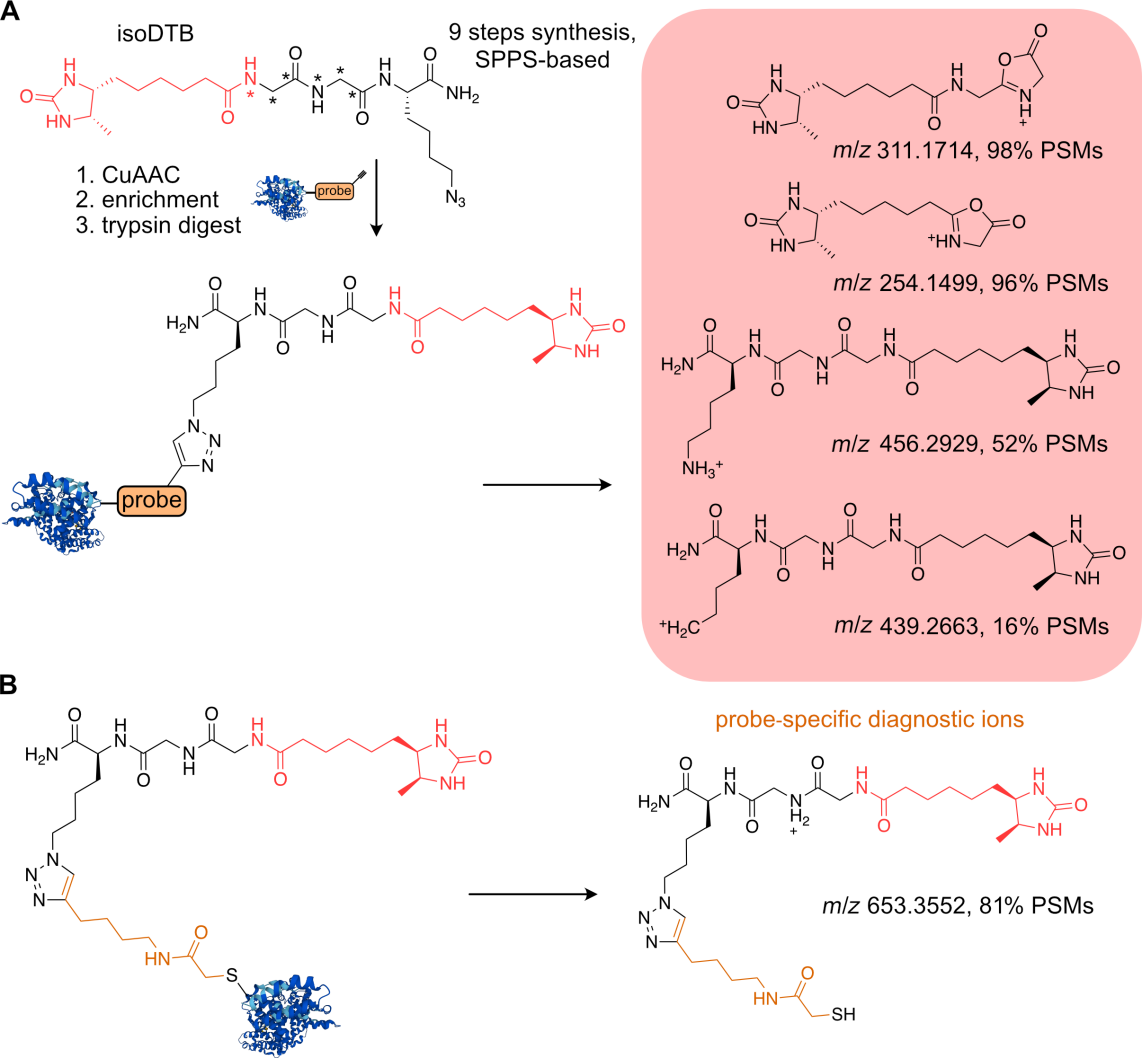
A) isoDTB linker and probe-specific diagnostic ions (B). *A star indicates the possible introduction of stable isotopes for MS quantification.

Recently, an isoDTB linker was introduced, which is synthetically easily accessible and now as well commercially available. IsoDTB combines the desthiobiotin moiety and isotopically labeled residues for direct quantification (Figure 9A) [103]. Thus far, the linker has been mainly utilized for profiling of reactive cysteines. For this review the labile and open search of the data acquired by Zanon et al. using the FragPipe suite led to identification of several anticipated diagnostic ions, including the b-type ions from fragmented peptide bonds of the linker (Figure 9A). The study builds on the reactivity of free cysteine thiols, which are reacted on the proteome-wide level with iodoacetamide-alkyne via S_N_ reaction. Similar as for the above-described DTB-linker, several probe-derived ions were observed (Figure 9B). The isoDTB-linker’s availability in ‘light’ and ‘heavy’ form together with highly optimized protocols and direct presets in MSFragger for streamlined analysis make this linker a feasible choice for site identifications of probe–peptide conjugates.

**Figure 10:**
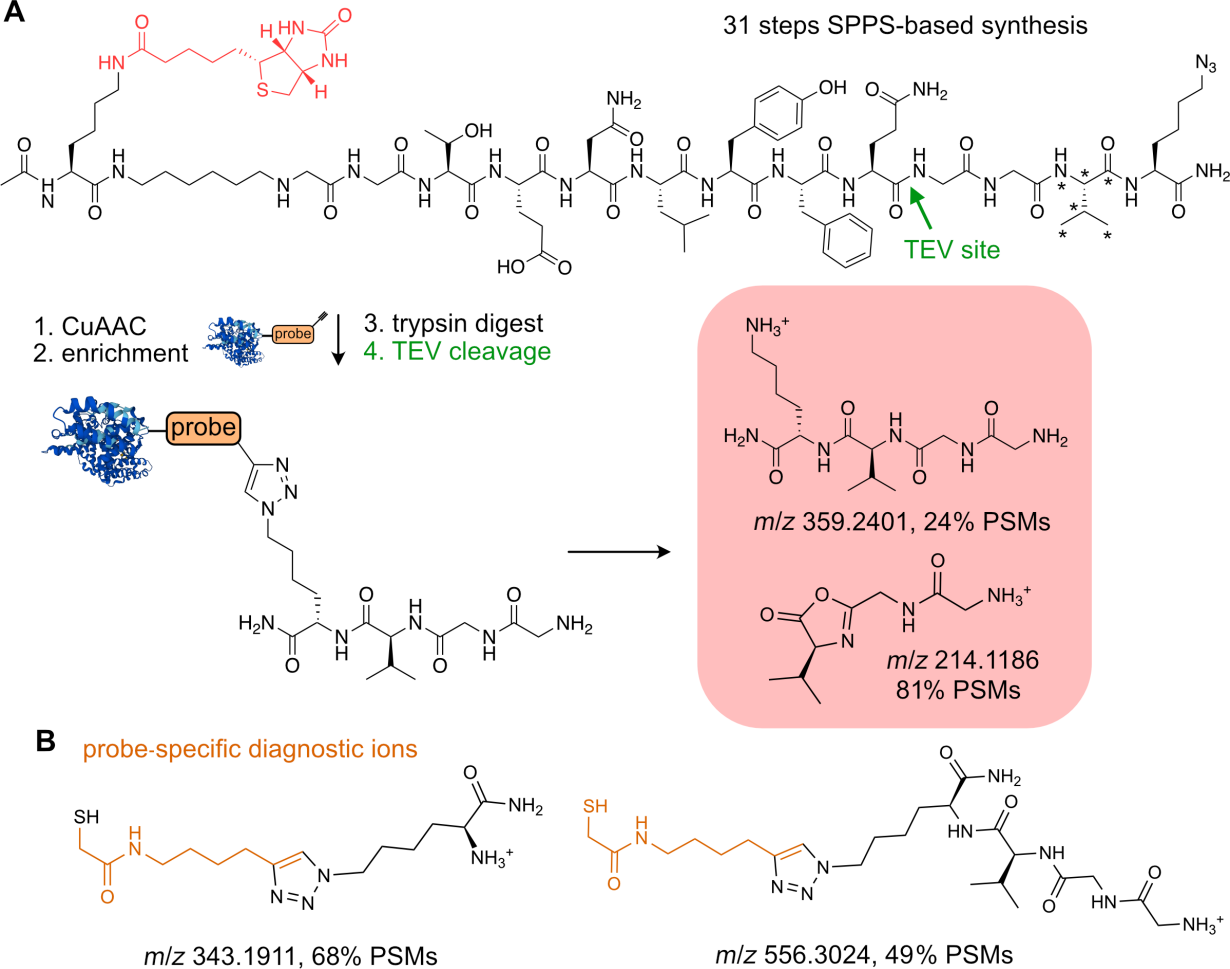
TEV-cleavable linker structure with its characteristic diagnostic ions (A) and probe-specific diagnostic ions (B). *A star indicates the possible introduction of stable isotopes for MS quantification.

**Figure 11:**
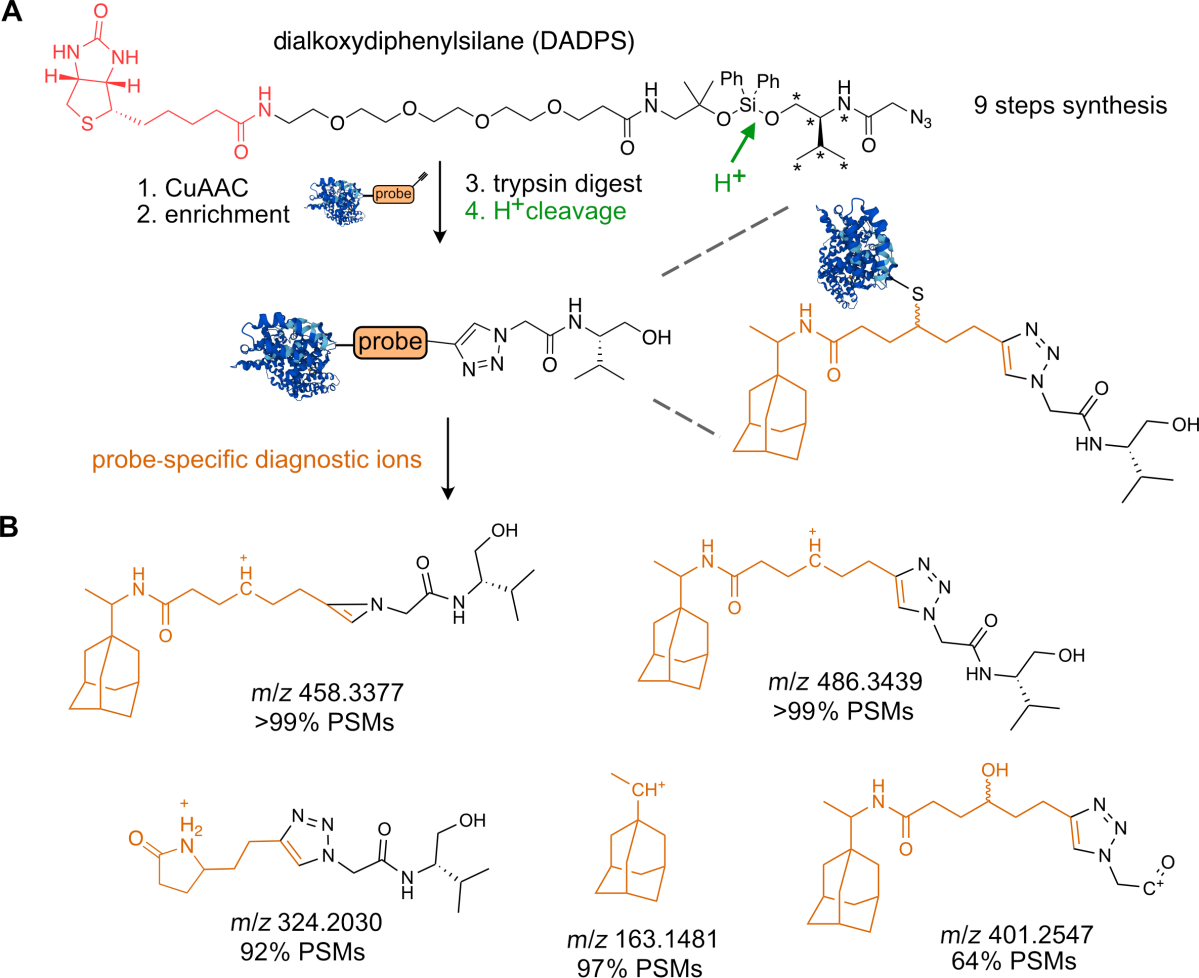
A) Structure of the full length DADPS linker and remaining part after cleavage. B) Diagnostic ions. *A star indicates the possible introduction of stable isotopes for MS quantification.

**Figure 12:**
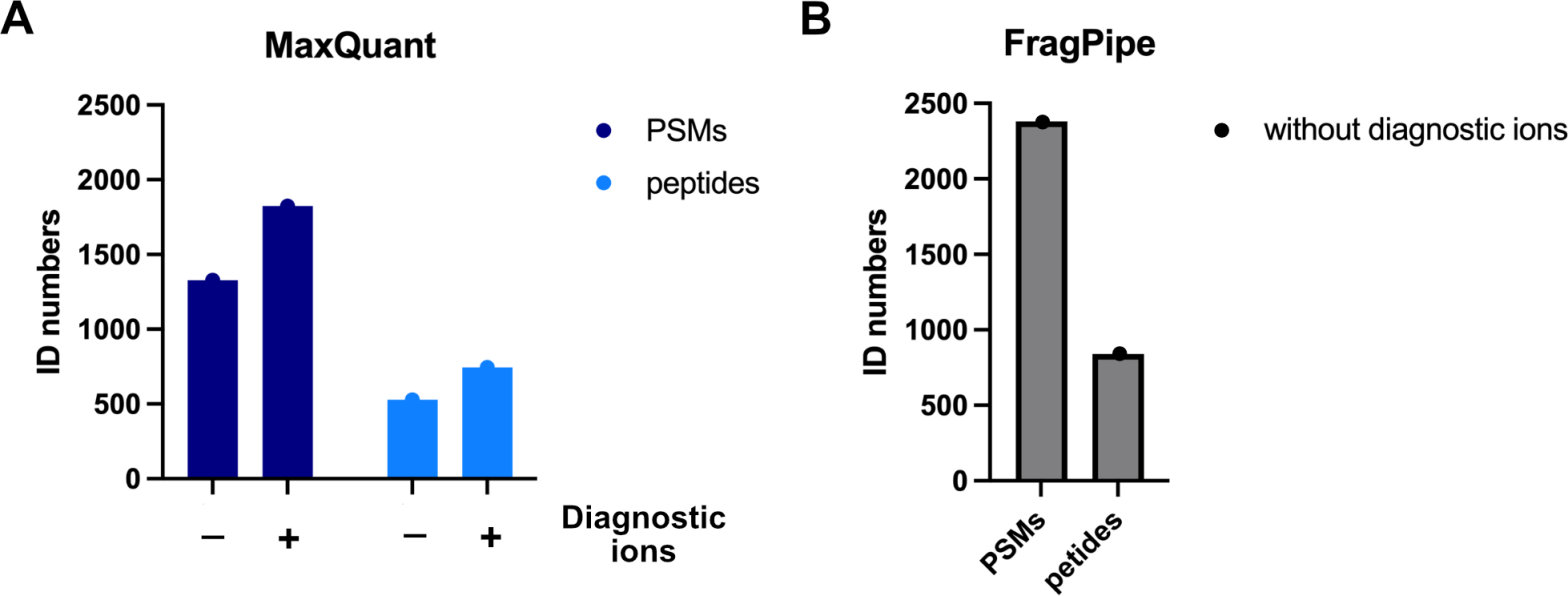
Diagnostic peaks included in the search identify higher numbers of modified PSMs and peptides using MaxQuant (A) and comparison with FragPipe (B) in case of the adamantane-based probe from the study by Wozniak et al. [110].

An alternative approach to selectively release the probe–peptide conjugates after the affinity enrichment is the utilization of a cleavable linker [52]. From the plethora of cleavable linkers reviewed by Beard et al., we focus here on selected few, which were used in recent studies with sufficient resolution and depth [52]. In general, cleavable linkers can be divided into two main groups based on the cleavage mechanism, which can be either enzymatic or chemical. The advantage of cleavable linkers is the possibility to combine stringent enrichment, ensured by the biotin–streptavidin complex, with the selective release of modified peptide–probe conjugates afterwards. The frequently used enzymatically cleaved TEV-linker contains a peptide sequence, which is recognized by Tobacco Etch Virus nuclear inclusion A peptidase, which is orthogonal to trypsin and chymotrypsin cleavage – two most commonly used proteases [109]. Multiple modifications to the peptide sequence and linker structure were consecutively introduced by the Cravatt lab including the ‘light’ and ‘heavy’ derivatives containing stable isotopes enabling the quantification and direct comparison between the conditions [80]. The disadvantage of the TEV-linker might be the multistep synthesis. The labile and open search of the publicly available data revealed a set of diagnostic ions, both derived from the linker and the probe (Figure 10A and B). The structural motives of the diagnostic ions corroborate the above-described findings. The fragmentation of the triazole ring leaving the primary amine and b-ion resulting from the fragmentation of the TEV-recognition peptide sequence. The chemical cleavage of the linker to release probe–peptide conjugates is achieved mainly by the change of the pH or via reducing conditions to release acid, base, or redox-labile parts of the linker [52]. Most recently, Wozniak et al. mapped the binding sites of a large small molecules library, which were equipped with a photo-crosslinker [110]. The study used the acid-cleavable dialkoxydiphenylsilane (DADPS) linker to release the probe–peptide conjugates (Figure 11A). The crucial advantage of the study was the introduction of a search algorithm considering generation of the chimeric spectra. The chimeric spectra may include fragmentation of multiple probe–peptide conjugates which differ only in the position of the probe–peptide covalent bond. Thus, the physicochemical properties are highly similar as well as the mass of the conjugate leading to coelution from LC and cofragmentation during MS/MS. The case study of the adamantane-based probe revealed at least five probe-derived diagnostic ions from labile search, which are summarized in Figure 11B. Next, we used the diagnostic ions in a closed search with the MaxQuant search engine. For this purpose, we set two calculation runs searching for peptides carrying a mass offset of 485.3366 on C, K, R, E, D, S, T, or H. The closed search including the diagnostic ions found ≈20% more identified peptides showing the search improvement by diagnostic ions (Figure 12). The DADPS-linker containing ‘light’ or ‘heavy’ valine can be synthetized in total nine steps [111].

**Figure 13:**
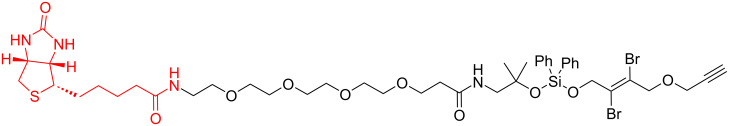
An alternative DADPS linker.

After the introduction of the DADPS-based linker by Szychowski et al. for the first time several interesting analogs were developed, including the shortened version described in the previous paragraph (Figure 13) [112–115]. As an interesting feature, the DADPS-linker with dibromo substitution takes advantage of the naturally occurring stable isotopes ^79^Br and ^81^Br in almost stoichiometric ratio. The predictable isotopic pattern thus introduces a searchable moiety and therefore clearly tags the MS/MS spectra derived from probe–peptide conjugates [113]. Another DADPS-based linker improves the CuAAC kinetics by using picolyl azide [115]. Together, the application of the acid-cleavable DADPS linkers is a feasible alternative to enzymatic cleavage. In particular, when considering the linker preparation, the biotin–DADPS–azide can be synthetized in only two steps [112].

**Figure 14:**
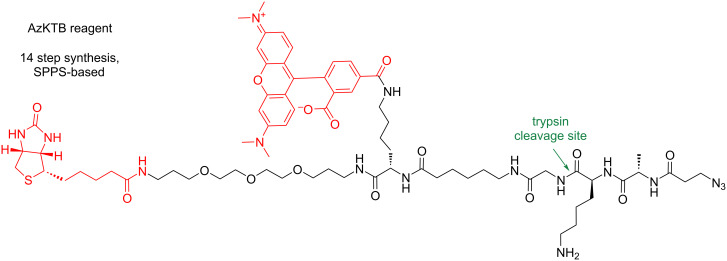
Chemical structure of the trifunctional trypsin cleavable AzKTB linker.

Finally, the development of multifunctional linkers enables to combine the in-gel and MS-based analysis in one experiment [116–120]. The trifunctional linker AzKTB (Figure 14) was introduced by Wright et al. to study protein *N*-myristoylation and was later used for the identification of the natural product callyspongynic acid and zerumbone protein targets [117,121–122]. The AzKTB linker integrates biotin for affinity enrichment with a TAMRA fluorophore for in-gel analysis, a terminal azide for CuAAC to label probe–protein conjugates, and a trypsin-cleavage site, which allows to release the modified probe–peptide conjugates together with other unmodified tryptic peptides from the streptavidin beads (Figure 14). In principle, the release of modified peptides can be used for side identification given the relatively high abundance and ionization efficiency due to remaining lysine. The use of the AzTB linker is practical for swift optimization of enrichment workflows, because after the probe–protein conjugates enrichment on the streptavidin beads, the beads can be split into two portions from which one is analyzed by in-gel analysis first to determine the overall labeling and the other portion after on-beads trypsin digest is subjected to LC–MS/MS analysis. The AzKTB reagent can be synthetized by standard solid-phase peptide synthesis (SPPS) with overall 17% yield.

In this review we would like to also highlight the importance of deposition of the acquired MS data to publicly available depositaries with a clear description of the sample preparation protocol. This may allow the community to research the data and apply the ever-improving search algorithms to find a new features and probe–protein interactions.

### Automatization of chemical proteomics workflows

The increased throughput and less hands-on time during the chemical proteomics workflows led recently to a number of protocols, which either multiplicate the sample preparation or scale down and partially automatize the protocol or both. The standard mass spectrometry-based chemical proteomics workflow requires up to 1 mg of total protein, acetone protein precipitation after the ‘click’ reaction to remove the excess of the biotin-containing reagent, desalting of the peptides after on-beads digest, and concentration of peptides either by freeze-drying or on SpeedVac. This standard protocol is typically carried out in individual 1.5 mL tubes which need to be handled separately. Indeed, the standard protocol would span over several days with a limited number of samples possibly processed in one batch. With the advancement of LC–MS/MS sensitivity and speed of spectra acquisition, it is possible now to scale-down the chemical proteomics protocol. There are several approaches with significantly increased throughput available for either probe–peptide conjugates [123–124] or probe–proteins enrichment [94]. The underlying and unifying advantage of these protocols is the use of a mixture of hydrophilic and lipophilic carboxylate-coated magnetic beads to replace the protein acetone precipitation after CuAAC. After the ‘click’ between the probe–protein conjugates and affinity tag in cell lysate, the proteins are loaded onto the carboxylate magnetic beads and aggregated with the beads by addition of an organic solvent, for example ethanol or acetonitrile. The subsequent washes of the carboxylate magnetic beads with proteins by an organic solvent–water mixture or organic solvent alone cleans the probe–protein conjugates from excess of ‘click’ reagents and non-protein components present in lysates with high efficiency. The fast sequence of the wash steps is performed on a magnet separating the magnetic beads with aggregated proteins from the wash solution. Next, the proteins can be proteolytically cleaved on-beads or directly eluted for further enrichment on an affinity resin. Due to the easy handling of the magnetic beads and separation for the liquid phase using the magnet, the protocol utilizing the carboxylate magnetic beads was first developed for whole proteome proteomics, so called single-pot solid-phase enhanced sample preparation (SP3) [125–126]. The workflow can be scaled down readily to low protein amounts and small volumes compatible with the 96 and 384-well plate format fitting to liquid handling robots. An automatization of the protocol indeed lowers the necessary hands-on time and improves robustness and reproducibility of the protocol. However, even the scale-down of the protocol into microplates with manual pipetting can significantly parallelize and thus speed up the process.

## Conclusion

In the future, the aim will be to unlock MS-based chemical proteomic workflows, which will obscure the necessity of an enrichment step and thus will lead to significantly simplified and accelerated characterization of important and desired probe–protein interactions. Until then, the transfer of expertise and techniques from different areas of the chemical proteomic field have the potential to facilitate this advancement.

## Data Availability

Data sharing is not applicable as no new data was generated or analyzed in this study.
